# Supplementary material for: Potentials and pitfalls of increasing prosocial behavior and self-efficacy over time using an online personalized platform
Source: PLoS One. 2020 Jun 25;15(6):e0234422. doi: 10.1371/journal.pone.0234422 (PMC7316236; doi:10.1371/journal.pone.0234422)
Supplement: Attachment — Submitted filename: Response to Reviewers.pdf [file pone.0234422.s001.pdf]

Opening Remarks:
We would like to thank the editor and our two reviewers for their constructive, careful and helpful feedback. We have tried to address all points made and believe the final manuscript to be stronger as a consequence of it. The greatest changes to the manuscript are the removal of the results and discussion of *likelihood to use*, an earlier and more thorough examination of self-efficacy theory, more detailed linking between theory and platform design, and a critical examination of the connection between platform design and research findings.

In the following are the point-by-point responses to all review points made.

**Point**	**Reviewer**	**Author Response**	**Corresponding sections and page number(s) in the revised manuscript**
Reviewer #1			
1	This is a well-written article on the effect of using a digital platform in prosocial behaviors. The theoretical background of the work clearly lays out each component of the research. However, **the two studied measures of self-efficacy (change impact and everyday helping) could have been introduced early on**	The introduction has been revised to introduce the two measures of self-efficacy early on.	Introduction (p. 3 in the revised manuscript)
2	The authors present a detailed description of the study design and developed platform components. As for the platform, it is stated that the goal of the research was to create a platform where participants can successfully master prosocial actions. **It is not clear, in designing the platform, how or why the critical components of the platform, such as personalized actions, gamified elements, and virtual rewards were selected. A strong justification for including these components in the platform would strengthen the article.**	A point-by-point justification for each included component (personalized actions and gamified elements, including virtual rewards) has been added to the section describing the platform.	Introduction (p. 5 in the revised manuscript)
3	Similarly, while details on the personalization of suggested actions were given, **such details for the other two components (gamification and virtual rewards) are lacking but critical for**	Details on the gamification (virtual rewards and map) have been added.	Method (p. 7-9 in the revised manuscript)

	**comprehending the merit of the work**.		
4	Furthermore, **information on how the COM-B model maps with these components would make the article stronger**. **For example, how is the capability, opportunity, and motivation of the users are known and integrated with the platform? Are these dynamic or static? Are these measures context-sensitive? Do these changes based on user activities over time?**	Information on how the COM-B model mapped to the components of the platform, including information on how we gained information on participants capability, opportunity, and motivation has been added. A discussion of the lack of context sensitivity has been added to the Limitation section.	Introduction (p. 7-9 in the revised manuscript) & Discussion (p. 25 in the revised manuscript)
5	In the study design, three variables (enjoyment, appreciation, usability) are measured to assess platform effectiveness. **The theoretical foundation or justification for using these variables should be included.**	The theoretical foundation for enjoyment, appreciation, and usability has been added in the *Platform experience* section.	Method (p. 12 in the revised manuscript)
6	A rigorous data analysis section adds value to the article. Adequate discussion is presented to share detailed insight. Limitations and further research address some of the critical aspects of the research. **In the Limitations section, authors should consider addressing how the design or execution of the platform may or may not have affected the findings. The team developed its own digital platform to carry out the research. It would be interesting to know if the design and implementation of the platform played any role in the findings**	A discussion of limitations created by the design and execution have been added and how the design and implementation played a role in the findings.	Discussion - Limitations (p. 25 in the revised manuscript)
7	As part of the research, the team developed a scale to measure everyday helping self-efficacy. This is a valuable contribution. **Design implications for fostering prosocial behavior through a digital platform is another valuable contribution of the work.**	Further discussion of the design implications have been added to the Discussion.	Discussion (p. 23-25 in the revised manuscript)
Reviewer #2			
8	This paper randomizes 66 psychology students (who is this case are experimental subjects who are interested in increasing	The discussion of self-efficacy has been revised to explain the nuances	Introduction (p. 2-3 in the revised

	their prosocial behavior) into two groups, Baseline and Platform. All subjects were asked to self-report their prosocial behavior for 3 weeks (daily measures and weekly), while subjects in the Platform treatment also had access to an online platform that recommends, gamifies, and rewards action. The main theoretical framework was that of self-efficacy - the belief that one's ability to successfully perform an action - will increase the likelihood that a behavior will be taken. The findings is that the platform has no effect on either the behavior or self-efficacy. If done convincingly, this is an interesting null finding and could be important, but there are other issues that weaken this study. **First, the paper does not discuss self-efficacy in a consistent way. For example believing that I have the discipline to put aside my money to donate is different from believing that the contribution will make a difference(p.2), enjoying the activity more (top of p.3), or developing more empathy (bottom of p.3). The connections between change impact self efficacy (p. 8), everyday helping (p.9) and general self efficacy (p.10) are not well developed.**	between these sections and how they tie into overarching self-efficacy theory.	manuscript)
9	**It is unclear whether including two of these measures result in multicollinearity (e.g in Table 6)...**	Centering the data, as we did, is used to stabilize and help prevent issues with multicollinearity in multilevel analysis (Field, Miles & Field, 2012). We have now rerun the analysis with the variables separately to compare results to the combined model, which did not lead to different results. A note on this is now included in the manuscript.	Results (p. 17 in the revised manuscript)
10	... and **[It is unclear] whether we expect a change in self efficacy to result in a change in behavior** (which suggest a different econometric model than what is	The theoretical background of self-efficacy and it's close relation to behavior has been further emphasized. As described in	Introduction (p. 2-3 in the revised manuscript)

	employed). I would suggest that the paper be refocused on self-efficacy, because right now the focus is too diffused.	previous points, the centrality of self-efficacy has now been further set into focus.	
11	Second, I think there is **just not enough data (or theoretical backing) to discuss Likelihood to use.** We have 33 people on the platform, with several dropped for being an outlier or not completing the survey, and we are splitting this further to Likely to use and Not Likely to use.	We agree that the sample discussed in the section on Likelihood of Use is small, but believe that Likelihood to Continue is important to discuss. We have therefore removed the results and discussion of the subsample analysis and refer only to the correlational results introduced in the first section on the Results.	Results and Discussion (p. 18, p. 22- 26 in the revised manuscript)
12	On the topic of not enough data, we have 8 people in post-interview...	The theoretical foundation behind the qualitative data analysis has been expanded to explore the reasoning behind the sample size chosen, which is in line with recommended sample sizes for such an interview analysis.	Results (p. 14-15 in the revised manuscript)
13	...which does not feel adequate for the claims that this paper is making, especially since the qualitative and quantitative results are different.	The discussion on the relationship between the quantitative and qualitative results has been extensively rewritten to reflect the synergistic potential of these methods.	Discussion (p. 22-23 in the revised manuscript)
Journal Requirements			
14	Please ensure that your manuscript meets PLOS ONE's style requirements, including those for file naming. The PLOS ONE style templates can be found at http://www.journals.plos.org/plosone/s/file?id=wjVg/PLOSOne_formatting_sample_main_body.pdf and http://www.journals.plos.org/plosone/s/file?id=ba62/PLOSOne_formatting_sample_title_authors_affiliations.pdf	The LaTeX Template provided on the PLOS ONE website was used and modified based on the provided style guide. Please let us know if other changes are requested.	Overall
15	We note that Table 3 may include questionnaire items that may have been previously published. The reproduction of previously published has implications for	All items were generated through the pilot study in this survey and have not been published in any previous publications. This has been added to	Method (p. 12 in the revised manuscript)

	the copyright that may apply to these publications. We would be grateful if you could clarify whether you have obtained permission from the original copyright holder to republish these items under a CC BY license. If you have not obtained permission to publish these items please remove them from your manuscript. You may wish to replace the text you have removed with relevant question numbers/ brief descriptions of each item.	the manuscript	
16	Please ensure that you include a title page within your main document. You should list all authors and all affiliations as per our author instructions and clearly indicate the corresponding author.	The title page with all authors, affiliation and corresponding author are now included.	Title page (p. 1 in the revised manuscript)
17	Please include captions for your Supporting Information files at the end of your manuscript, and update any in-text citations to match accordingly. Please see our Supporting Information guidelines for more information: http://journals.plos.org/plosone/s/supporting-information.	Captions for the supporting information files have been added to the end of the manuscript.	Supporting Information (p. 32 in the revised manuscript)

References

Field, A., Miles, J., & Field, Z. (2012). *Discovering statistics using R*. Sage publications.
